# Lipopolysaccharides: From Erinyes to Charites

**DOI:** 10.1155/2012/684274

**Published:** 2012-05-14

**Authors:** Alfredo Focà, Maria Carla Liberto, Angela Quirino, Giovanni Matera

**Affiliations:** Department of Health Sciences, Institute of Microbiology, University “Magna Graecia” of Catanzaro, Via T. Campanella, 115, 88100 Catanzaro, Italy

## Abstract

Following the discovery of endotoxins by Richard Pfeiffer, such bacterial product was associated to many severe disorders produced by an overwhelming inflammatory response and often resulting in endotoxic shock and multiple organ failure. However, recent clinical and basic sciences investigations claimed some beneficial roles of typical as well as atypical endotoxins. The aim of this paper is to focus on recent data supporting a beneficial activity of both typical and atypical endotoxins. Such novel perspective looks promising for development of new drugs for prevention and therapy of several human diseases.

## 1. Introduction

The Erinyes were three netherworld goddesses depicted as ugly, winged women with hair, arms, and waists entwined with poisonous serpents and personified the tormenting madness inflicted upon a patricide or matricide. They could make people suffer, and a nation harbouring such people could experience dearth, and with it hunger and disease. On the contrary, the Charites, also commonly known as the Graces, were three goddesses daughters of Zeus and named Aglaia, Thalia, and Euphrosyne. They were often associated with grace, beauty, adornment, mirth, festivity, dance, and songs of revel [1].

The endotoxins of Gram-negative bacteria are lipopolysaccharides (LPSs), which are vital to both the structural and functional integrity of the bacterial outer membrane [2, 3].

In the first reports on endotoxin by Pfeiffer (1892) and Centanni (1894), only one side of the coin (the toxic activity) has been considered [4]. Lewis Thomas reported the reaction of higher animals (including humans) to endotoxins as “… a uncontrolled and auto-destructive behaviour of the host, leading to the consideration of endotoxin as a venom. All this seems unnecessary, panic-driven. There is nothing intrinsically poisonous about endotoxin, but it must look awful, or feel awful, when sensed by cells …” (Lewis Thomas, Germs, 1974). However, endotoxins may behave not only as Erinyes but also as Charites. Indeed many LPS activities are being increasingly revealed to be beneficial to the host. Some of such beneficial activities have been published a few years after endotoxin discovery, including the inhibitory effects on human sarcoma studied (since late 1890s) by William Bradley Coley, who used killed *Serratia marcescens*, and the successful therapy of lethal tertiary syphilis reported by the 1927 Nobel laureate Julius Wagner von Jauregg, who used different types of microbial suspensions [4].

The purpose of this paper is to focus on recent data supporting beneficial activities of both typical and atypical endotoxins.

## 2. Chemistry of Typical and Atypical Lipopolysaccharides

The lipid A, the core oligosaccharide, and the O-antigen polysaccharide chain are the three domains usually found in an LPS molecule.

The innermost, hydrophobic region, lipid A, is responsible for the major toxic and beneficial properties of bacterial endotoxins [2]. Lipid A is the least variable part of the molecule among the different species of a genus, and its structure generally consists of a diglucosamine backbone substituted with varying numbers (usually four to seven) of ester- or amide-linked fatty acids.

**Figure 1:**
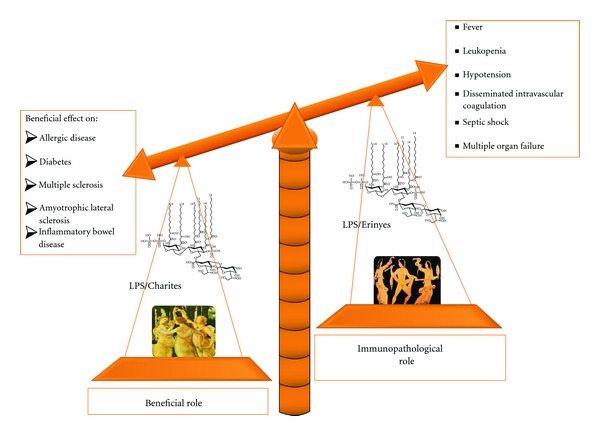
Balance between beneficial and immunopathological roles of LPSs.

Phosphate and/or other substituents are linked to carbons at the C-1 and C-4′ positions of the glucosamine disaccharide [3] (Figure 1). A 2-keto-3-deoxyoctonate (Kdo) unit links the lipid A to a core oligosaccharide (OS) composed of about 10 sugar residues. The core is linked to a third outermost region of a highly immunogenic and variable O-chain polysaccharide (PS) or O-antigen made up of repeating OS units. The latter region of the LPS molecule is responsible for bacterial serological strain specificity and is present only in smooth-type bacteria. The core region of enterobacterial LPS includes an outer portion, distal from lipid A (proximal to the O-polysaccharide chain), and an inner portion directly linked to the lipid A. The complete outer core region (Ra-structure) mainly consists of hexoses and hexosamines, whereas inner core region is composed of KDO and heptose. The so-called rough-type bacteria produce LPSs lacking O-antigens [2, 3]. A successive truncation of Ra-structure LPSs associated with specific alterations of core oligosaccharide biosynthesis in different *Salmonella *strains (R-mutants) results, respectively, in the Rb, Rc, Rd, and Re core structures. The last structure, which contains only lipid A and KDO residues, is a minimal LPS structure. The lipooligosaccharides (LOSs) consist of lipid A and an oligosaccharide core. The structural organization of LOS allows us to assign them to the group of intermediate molecules between typical R- and S-LPS structures.

The lipooligosaccharides (LOSs) contain a recognizable, well-conserved inner core (including KDO and heptose residues) from which extend one or two/three mono- or oligosaccharide branches (such as *α*-, *β*-, and *γ*-chains in Neisseria LOSs), that exhibits serological specificity [3]. In classical LPSs, the core provides an acceptor for O-polysaccharide, on the contrary in LOSs (distinct from R-LPS) the core is destined to terminate without O polysaccharide addition [2, 3].

LOSs are identified in such Gram-negative bacteria as *Bordetella pertussis*, *Neisseria meningitidis*, *Neisseria gonorrhoeae*, *Haemophilus influenzae*, *Haemophilus ducreyi, Burkholderia (Pseudomonas) multivorans, Burkholderia (Pseudomonas) cenocepacia, Alteromonas addita* KMM 3600T, and *Campylobacter jejuni *[2, 3].

Atypical LPSs reportedly exhibit a lipid A chemistry which is different from archetypal structure found in *Escherichia coli* and *Salmonella*. Namely, atypical lipids A from different bacteria have the same general structure, but differ in the head-group substituents (e.g., phosphate groups) and in the number, distribution, and composition of fatty acids [2, 3].

In addition to the presence of fatty acids with hydrocarbon chain longer than 14 carbon atoms, the charge of lipids A from *Helicobacter pylori, Porphyromonas gingivalis, Francisella tularensis* was lower than the charge of lipids A from *E. coli* and compound 506, which could also affect the binding. The low affinity of LPS binding with LBP and/or sCD14 is likely to influence the rate of endotoxin delivery to membranes of target cells and as a result to decrease the effectiveness of LPS signalization [2].

Matera et al. [5] reported that *Bartonella quintana* LPS exhibited a migration pattern of the deep rough chemotype.

*Bartonella henselae* has been found to exhibit a deeply atypical LPS with an approximate molecular weight of 5000 and with a Lipid A containing an acyloxyacyl residue 16:0[3-O(28:0(27-OH))] [6].

Therefore, LPS of *Bartonella henselae* has a deep-rough structure without an O-chain polysaccharide and contains an unusual penta-acylated lipid A with a long-chain fatty acid. The absence of O-side chain could conceivably decrease complement fixation and provide a degree of serum resistance on *Bartonella*, but this possibility has not been explored. The unusual fatty acid composition renders *Bartonella henselae* endotoxin at least 1000-fold less potent at Toll-like receptor (TLR)4 activation (as measured by IL-8 production), as compared with LPSs from *Salmonella *[3, 6].

 LPS also serves as one of the primary targets of the innate arm of the mammalian immune system, whose Toll-like receptors (TLRs) are the primary Pathogen Recognition Receptors (PRR). A wealth of publications indicated TLR4 and TLR2 as the receptors involved in the recognition of most of the LPS studied [2, 3, 6, 7].

LPSs are known as endotoxins, which cause the prominent pathophysiological symptoms associated with sepsis and septic shock, that is, fever, leukopenia, hypotension, disseminated intravascular coagulation, and multiple organ failure [2, 3]. The well-known typical LPS from enteric bacteria, such as *Escherichia coli* and *Salmonella enterica*, are highly potent molecules with regard to their biological, that is, endotoxic activities [6].

## 3. Beneficial Activities of Typical LPSs

Naturally occurring (often typical) LPSs modulate the immune system of higher vertebrates in order to keep pathogens away and to avoid the possibility of saprophytes/commensals to become invaders (translocation); moreover, it has been demonstrated that the immune system is dependent on certain microbial products including LPSs for normal development [7].

Epidemiology studies in young children have found that LPS exposure at home is inversely correlated with the development of atopic diseases, following the “hygiene hypothesis” for allergic disorders [8].

The growing prevalence of broadly diffused chronic, inflammatory, and degenerative diseases in the industrialized world (allergic illnesses, diabetes and other metabolic disorders, inflammatory bowel diseases and, within the central nervous system (CNS), demyelinizing inflammatory pathologies, as well as stroke) might ask for a broadening of such “hygiene hypothesis” [9], which should also include the above reported chronic/inflammatory diseases [10].

In an asthma model, nonobese diabetic (NOD) mice were immunized intraperitoneally on day 0 with ovalbumin (OVA) in presence of alum, challenged one week later with 3 consecutive OVA aerosol administrations and analyzed 24 hrs after the last challenge. Following this protocol, mice presented allergic inflammation and abnormal lung function. Allergic inflammation resulted in an increase of cell recruitment including eosinophils in the BALF, and of cytokine and chemokine production, IL-4, and eotaxin, respectively, in the lung. Mice treated with TLR agonists, particularly LPS, showed a decreased eosinophilia and IL-4 and eotaxin production as compared to control mice [11].

In a NOD mice experimental model, the effect of TLR ligands, including LPSs, on development of spontaneous diabetes was evaluated. In NOD protected (LPS-treated) animals, the histological analysis of the pancreas showed a reduction in destructive islet infiltration (i.e., invasive insulitis). This form of insulitis is associated with active destruction of insulin-secreting *β*-cells; this is the point in time, where the first mice showing overt hyperglycemia can be seen. It appears that in the case of LPS treatment a control of insulitis progression and hyperglycemia can be observed [11, 12].

To address the intricate relationship between gut microbiota and host cells, colitis was induced in C57BL/6J mice with dextran sodium sulfate (DSS) or by transferring CD45Rb(hi) T cells into RAG1^−/−^ mice. Colitis severity was assessed by disease activity index (DAI) and histology. The effect of anti-TLR4 antibodies (Ab) on the inflammatory infiltrate was determined by cell isolation and immunohistochemistry. Mucosal expression of inflammatory mediators was analyzed by real-time PCR and ELISA. Blocking TLR4 at the beginning of DSS administration delayed the development of colitis with significantly lower DAI scores. Anti-TLR4 Ab treatment decreased macrophage and dendritic cell infiltrate and reduced mucosal expression of CCL2, CCL20, TNF-alpha, and IL-6. Anti-TLR4 Ab treatment during recovery from DSS colitis resulted in defective mucosal healing with lower expression of COX-2, PGE(2), and amphiregulin. In contrast, TLR4 blockade had minimal efficacy in ameliorating inflammation in the adoptive transfer model of chronic colitis. Therefore, anti-TLR4 therapy may decrease inflammation in IBD but may also interfere with colonic mucosal healing [12].

Deficient TLR signaling may cause an imbalance in commensal-dependent homeostasis, facilitating injury and leading to inflammatory bowel disease. Accordingly, systemic administration of a TLR4-blocking antibody impairs restoration of tissue integrity during DSS-colitis, despite limiting exaggeration of acute inflammatory responses induced by recruited cells. Several recent studies suggest that TLR signaling exerts many important cytoprotective functions in the intestinal epithelium (and adjacent cell subsets), which are required for barrier preservation, cell survival and stability, and restitution, including, for example, inhibition of apoptosis, migration, and proliferation [13, 14].

Thus TLR4 agonists such LPS could be beneficial in colonic mucosal healing during IBD.

Animals exposed to LPS as neonates displayed induction of IL-10 within the CNS, and there was a robust inverse correlation between experimental autoimmune encephalomyelitis severity and the frequency of CNS-infiltrating FoxP3^+^ T lymphocytes. These observations were supported by reduced FoxP3 expression in brain tissue from multiple sclerosis (MS) patients compared with non-MS patients [15].

A small dose of LPS given systemically confers ischemic protection in the brain, a process that appears to involve activation of an inflammatory response before ischemia. LPS preconditioning in the brain shares some hallmarks that are characteristics of ischemic preconditioning in other organs. Interestingly, it has been reported that pretreatment of animals with LPS increases myocardial functional recovery in ischemia/reperfusion heart injury model. Such LPS-induced beneficial effect has been shown to be mediated through inhibition of NF-*κ*B via increase of HSP70. These include delayed induction of tolerance after preconditioning and dependence on “de novo” protein synthesis. The systemic route of LPS administration and the induction of some systemic changes are unique aspects of LPS preconditioning that might offer some clinical advantages [14–16]. Also, the very recent paper by Mouihate et al. [17], underlined that early postnatal LPS exposure remodulates neuroimmune axis allowing enhanced activation of a novel prostaglandin-mediated activation of the hypothalamic-pituitary-adrenal (HPA) axis brought about by increased constitutive expression of TLR4 and COX2 [17]. Reprogramming the neuroimmune axis during infancy might be beneficial in the rest of animal and human life. Such LPS-driven tight regulation of overwhelming or inappropriate immune system activation would pay off during acute systemic inflammatory reaction (e.g., sepsis/septic shock) or severe allergic disorders (e.g., asthma attack) in adult life.

## 4. Beneficial Effects of Atypical LPSs

Some bacteria (e.g., *Bartonella, Yersinia, Rhodobacter, Chromobacterium*) contain an atypical LPS with low endotoxic activity and/or prominent antagonistic effect on LPSs from enteric bacteria [2, 17–19].

Coevolution of organisms bearing a deeply modified/atypical LPS with a vertebrate host would be beneficial to both of them. Indeed the microorganisms factors including LPS may reduce/inhibit the inflammatory potential of same tissue/district (respiratory and digestive mucosal, CNS).

Rough mutants of *Yersinia enterocolitica* exhibited atypical LPS and attenuated virulence and lack of ability to colonize organs as spleen and liver. Even more interestingly such mutants showed a substantial impairment of several other virulence factors, which depend on a full structure of LPS for proper function and/or expression [20]. Therefore, these strains might be exploited for preparation of vaccines or adjuvants.

Similarly to other atypical LPS-bearing bacteria also *Bartonella spp.* are endowed with anti-inflammatory activities which might be exploited for medical purposes [18, 19].

*Bartonella spp*.LPS have been found to behave in a manner that is substantially different from other LPSs from saprophytic, commensal, and pathogenic microorganisms.

Matera et al. [5] reported that *B. quintana* LPSs exhibited a migration pattern of the deep rough chemotype, a strong reactivity following the chromogenic Limulus amoebocyte lysate test and a very low cytokine release from human whole blood samples. In human leucocytes or in endothelial cells [21], as well as in a rat model [22], *B. quintana *LPS was not able to induce significant levels of blood TNF*α*. Moreover, *B. quintana *LPS induced an increase in the white blood cell count without a substantial change in heart rate, hematocrit, platelet count, or blood pressure [22]. Remarkably, *Bartonella quintana *LPS possesses antagonistic properties for TLR4 and does not activate TLR2 [18, 19]. However, the physical-chemical features of *B. quintana* LPS warrant further investigations for a more in-depth knowledge of the structure-activity relationship.

The atypical LPS attributes undoubtedly contribute to the establishment and maintenance of mild although persistent infection, since the bacterium's major surface component is subinflammatory and antagonistic to the host's innate immune response. Interestingly, long-chain fatty acids are a conserved feature in the LPS of intracellular bacteria that establish long-term symbioses with their host, including *Legionella, Chlamydia*, and closely related rhizobia [2].

The control of inflammatory illnesses and the decrease of allergic/atopic disorders might be obtained by the administration of such antagonistic LPS species. Also the control of experimental rheumatic disease has been obtained by administration of TLR4 antagonist LPS from *B. quintana *[23]. However, impaired NF-*κ*B translocation by LPS pretreatment was also observed in TLR4-transfected overexpressing cells, suggesting that downregulation of TLR4 or TLR4 antagonism are not necessary events in impaired signal transduction in LPS-tolerant cells/tissues [24] and pointing to downstream site(s) of regulation and control of TLRs-dependent cascades carried out by LPSs and other bacterial products [7].

The complex population of microbes that we harbor within our mucosal cavities is not just passive bystanders, rather these organisms seem to actively shape our immune system responses both along the mucosal surface and in very remote tissues/organs [11].

Therefore, we suggested that some pivotal virulence factors, such as LPSs, control broad and increasingly diffused chronic, inflammatory, and degenerative diseases during the human evolution [25].

More interestingly some atypical LPSs could be plausible candidates to be developed into useful drugs for many diseases such as allergic illness, inflammatory bowel disease, and demyelinizing pathology of CNS [25].

## 5. LPS Derivatives as Adjuvants and Vaccines

 Furthermore, enzymes involved in Lipid A biosynthesis/modification [3] not only provide access to new lipid A derivatives that may be useful as adjuvants or endotoxin antagonists, but also can be exploited for generating novel live bacterial vaccines. Heterologous expression of lipid A modification enzymes like LpxE, LpxF, LpxR, or PagL in pathogens such as *Salmonella* might attenuate these bacteria by altering lipid A structural elements recognized by the TLR-4/MD2 complex [3].

Monophosphoryl lipid A (MPL) has been obtained from *Salmonella minnesota* R595 by removal of core KDO, one phosphate and one acyl chain from disaccharide backbone. MPL is among the recently licensed adjuvants and is used in combination with alum in recently approved vaccines for human papillomavirus and hepatitis B virus. Adjuvants can modify the delivery of the antigen or act as immunopotentiators, influencing both the amount and the quality of the adaptive immune response. Delivery can be modified through the slow release of antigen and enhancement of uptake by APCs in emulsions and liposomes, for example, whereas immunopotentiators act through the activation of the innate immune system [26].

## 6. Conclusions

While it seems clear that the microbiota influences progression and/or prevention of disease, the mechanism by which it can accomplish this task remains to be assessed. We have presented evidence that there is an intimate relationship between host and microbe that involves bacterial LPSs and host intricate mechanisms.

As many other molecules in biology, LPSs appeared as a “double-edged sword” [19]. Beneficial activity of both typical and atypical endotoxins look promising for the development of new drugs for prevention and the therapy of several human diseases.
